# Quality of Life Assessment in Romanian Patients with Spinal Muscular Atrophy Undergoing Nusinersen Treatment

**DOI:** 10.3390/neurolint16050067

**Published:** 2024-08-26

**Authors:** Bogdana Cavaloiu, Iulia-Elena Simina, Lazar Chisavu, Crisanda Vilciu, Iuliana-Anamaria Trăilă, Maria Puiu

**Affiliations:** 1 PhD School, Faculty of Medicine, Department of Microscopic Morphology, Genetics Discipline, Center of Genomic Medicine, ‘Victor Babeş’ University of Medicine and Pharmacy of Timișoara, 300041 Timisoara, Romania; bogdana.cavaloiu@gmail.com; 2 Department of Radiology, “Victor Gomoiu” Children’s Clinical Hospital, 022102 Bucharest, Romania; 3 Department of Genetics, Center of Genomic Medicine, ‘Victor Babeş’ University of Medicine and Pharmacy of Timișoara, 300041 Timisoara, Romania; maria_puiu@umft.ro; 4 Nephrology Department, University of Medicine and Pharmacy “Victor Babes”, 300041 Timisoara, Romania; chisavu.lazar@umft.ro; 5 Centre for Molecular Research in Nephrology and Vascular Disease, Faculty of Medicine “Victor Babes”, 300041 Timisoara, Romania; 6 Department of Neurology, ‘Carol Davila’ University of Medicine and Pharmacy, 020021 Bucharest, Romania; crisanda.vilciu@umfcd.ro; 7 Neurology Clinic ‘Fundeni’ Clinical Institute, 022328 Bucharest, Romania; 8 Department of Pathology, ‘Pius Brinzeu’ Emergency County Clinical Hospital, 300723 Timisoara, Romania; trailaiuliana@gmail.com; 9 Regional Center of Medical Genetics Timiș, Clinical Emergency Hospital for Children “Louis Țurcanu”, 300011 Timisoara, Romania

**Keywords:** spinal muscular atrophy, quality of life, SF-36, nusinersen

## Abstract

Spinal muscular atrophy (SMA), identified over a century ago, is characterized by severe muscle wasting and early mortality. Despite its rarity, the high carrier frequency of the responsible genetic mutations and the variability in its manifestations make it a significant research focus. This prospective cross-sectional descriptive study evaluated health-related quality of life (HRQoL) across eight health domains in 43 Romanian SMA patients treated with nusinersen, using the SF-36 questionnaire to analyze influencing factors. The survey was conducted online with informed consent, and the data were analyzed using MedCalc software, employing both parametric and non-parametric statistical tests for accurate interpretation. The results revealed significant variations in HRQoL. Most patients were non-ambulatory (74.4%), reflecting SMA’s impact on mobility. Urban residents reported better outcomes, particularly in physical functioning (*p* = 0.014), which may be attributed to improved access to healthcare services. Younger participants (under 14), represented by proxy responses, noted better general health (*p* = 0.0072) and emotional well-being (*p* = 0.0217) compared to older participants. These findings suggest that younger patients or their proxies perceive a better health status, highlighting the need for age-specific approaches in SMA management and the potential optimistic bias associated with proxy reporting on perceived health outcomes.

## 1. Introduction

First described at the end of the 19th century [1,2], spinal muscular atrophy (SMA) is a rare degenerative neuromuscular disease affecting alpha motor neurons found in the anterior horns of the spinal cord, characterized by progressive weakness and wasting of muscles, particularly in the proximal areas of the body, loss of ambulation, respiratory failure, and premature death [3]. It has a high carrier frequency ranging from 1:16 to 1:83, with a mean of 1:50, which makes it among the most commonly inherited autosomal recessive disorders, occurring at an estimated incidence of 1 in 6000–10,000 live births [4].

SMA is primarily caused by mutations or deletions in the survival motor neuron 1 (SMN1) gene found on chromosome 5q13.2, leading to insufficient SMN protein levels [5,6,7].

SMA is divided into four main subtypes based on severity: Type 0: Most severe with generalized weakness, hypotonia, respiratory distress, and poor feeding in newborns with one SMN2 copy [8]. Type 1: Classic SMA, appearing in 0–6 months with limb weakness and respiratory issues, expected in 45% of cases, and survival rarely beyond two years [7]. Type 2: Begins 6–18 months with three SMN2 copies, allowing sitting but not standing or walking. Respiratory problems and dysphagia are typical, with 68.5% surviving to age 25 [9]. Type 3: Starts between 18 months to adulthood, affects 15% of cases, and allows initial mobility but deteriorates over time. Divided into Type 3a (18 months to 3 years) and 3b (3 to 30 years) [8]. Type 4: Begins at age 30 or older, with most maintaining walking ability [10].

SMA management traditionally included therapies; a breakthrough was the FDA’s 2016 approval of nusinersen [11]. Nusinersen, an antisense oligonucleotide, corrects SMN2 exon 7 splicing to increase functional SMN protein production, improving motor symptoms. Additionally, two other high-cost and effective treatments exist, Onasemnogene Abeparvovec and Risdiplam [12]. Current studies focus on these medications’ use, selection, monitoring, and future research and health improvements in treated patients [13,14,15,16,17,18,19,20,21,22,23,24,25,26].

Various groups worldwide have extensively surveyed the health-related quality of life (HRQoL) in SMA patients, excluding the Romanian population. In 2008, Romania enhanced rare disease management by forming the National Alliance of Rare Diseases, uniting specialists and patients. Since October 2018, SMA associations and specialists secured nusinersen access for SMA patients through the National Program for Rare Disorders.

This study assessed health-related quality of life in Romanian SMA patients on nusinersen, exploring influencing factors with the SF-36 survey (36-Item Short Form Survey). Only one previous study has approached HRQoL in Romanian patients with SMA [27]. This study utilized the PedsQL-Family Impact Module (PedsQL-FIM) and the Cognitive Emotion Regulation Questionnaire (CERQ) to examine mothers of children with SMA, investigating the potential relationship between quality of life domains and corresponding emotion regulation strategies.

## 2. Materials and Methods

### 2.1. Study Design and Participants

This prospective cross-sectional descriptive study was conducted at “Victor Babes” University of Medicine and Pharmacy Timisoara, Romania, over six months from August 2021 to January 2022, with ethics committee approval No. 83/10 September 2021, revised 2024. Initially designed as in-person interviews, the COVID-19 pandemic necessitated a shift to an online study format. Inclusion criteria required a genetically confirmed diagnosis of spinal muscular atrophy independent of age, treatment with nusinersen, and the ability to complete the study questionnaire, even with proxy assistance. The survey was administered to 43 patients identified with type 1, 2, or 3 SMA or to their parents or guardians for children under 14. No personal data were collected, ensuring participant confidentiality and compliance with ethical standards.

### 2.2. Health-Related Quality of Life Assessment

The SF-36 questionnaire is a widely used tool for measuring quality of life. It consists of 36 straightforward questions grouped into eight domains: physical functioning, energy/fatigue, emotional well-being, physical and emotional limitations in work and daily life, bodily pain, and general health. This instrument has been employed to evaluate the quality of life for our cohort [23,28,29].

Participants received the survey and several additional questions via an electronic link using the Google^®^ Forms platform (Google Inc., Mountain View, CA, USA), with digital monitoring conducted by the authors. An introductory statement explained the study’s purpose and assured data anonymity. Participants provided informed consent by clicking “I agree.”

The questionnaire was divided into three sections:

- Three socio-demographic questions (age, sex, rural/urban residence).
- Six questions on factors influencing quality of life (SMA type, SMN2 copies, age at diagnosis, tube feeding, and ventilator use).
- The original 36 items from the SF-36 survey.

Scoring the SF-36 survey is a systematic process that involves two steps. Initially, responses to the survey items are recoded according to predefined percentage values (0 to 100 range), with higher scores representing better health. After recording, the next step involves averaging the scores for items within the same category to compute scale scores, meaning that individual items related to physical functioning or emotional well-being are combined to provide an overall score for each health dimension. Missing data are excluded from scale score calculations [28,29].

Regarding age considerations, the SF-36 survey was initially validated for individuals over 18 and was gradually adapted for use at ages 16, 14, and 12. For younger children, “proxy reporting” is used where parents or guardians provide information on behalf of the children.

The SF-36 survey includes robust mechanisms for handling responses, ensuring derived scores accurately reflect available outcomes without imputation. This approach simplifies data processing and enhances result reliability, making the SF-36 survey a valuable tool for clinical assessments and research in diverse healthcare settings.

### 2.3. Statistical Analyses

The statistical analysis was conducted using MedCalc^®^ Statistical Software version 22.021 (MedCalc Software Ltd., Ostend, Belgium; https://www.medcalc.org; 2024, accessed on 24 May 2024), ensuring reliable statistical processes. A *p*-value of less than 0.05 was considered statistically significant throughout the analysis, guiding the interpretation of the data. The analysis utilized different tools and tests to evaluate data involving different variables. Continuous variables were first tested for normality using the Shapiro–Wilk test. Normally distributed variables are described with means and standard deviations and were analyzed using the *t*-test or ANOVA where appropriate. Non-normally distributed variables are expressed as medians with interquartile ranges and were analyzed using non-parametric tests such as the Mann–Whitney or Kruskal–Wallis tests, depending on the number of tested groups.

## 3. Results

### 3.1. Demographic Analysis

We observed a right-skewed age distribution in our cohort of 43 patients (ages between 3 and 72 years old), with 13 patients under 14 years of age (30%) and 30 patients over 14 years old (70%). The mean age of the participants is 22.52 years, indicating a relatively young cohort. This is further supported by the median actual age, which at 20 years suggests that half of the participants are 20 years old or younger. The IQR—the 25th percentile at 9 years and the 75th percentile at 30.5 years—demonstrates a wide age spread among participants. The standard deviation of 15.79 years underscores the high variability in age within the cohort. The Shapiro–Wilk test for normality, with a test statistic of 0.914 and a *p*-value of 0.0034, indicates that the age distribution does not follow a normal distribution (Table 1).

The age at diagnosis reveals an average age of 2.953 years, with a median of 2 years and a range from 2 to 3 years. This indicates that half of the cases were diagnosed around the age of 2 years, with a somewhat narrow spread. The standard deviation of 2.225 years suggests variability in diagnosis age among different cases, but this is still relatively focused on early childhood. The Shapiro–Wilk test result of 0.697 with a *p*-value less than 0.001 strongly suggests that the age at diagnosis does not follow a normal distribution.

This research revealed several categorical variables associated with SMA, such as type, the number of SMN2 copies, geographical location (urban vs. rural), feeding tube requirement, and ventilator dependency (Table 1). Most individuals have Type 2 SMA, accounting for 60.4% (26 out of 43); Type 3 accounts for 27.9%, and Type 1, the most severe form, is less common at 11.6%. A majority, 67.4%, have 2 SMN2 copies, indicating a higher likelihood of severe phenotypes, as fewer copies correlate with disease severity. Those with three copies represent 32.6% of the sample. Across geographical distribution, 69.8% of the sample resides in urban areas. A total of 51.2% require respiratory assistance with a ventilator. This split suggests a balanced need for ventilatory support among the participants, reflecting the variable impact of SMA on respiratory function.

### 3.2. Baseline Characteristics of Dataset Stratified by SMA Type

The data from our research across SMA Type 1, Type 2, and Type 3 reveal significant variability in clinical presentations and treatment needs influenced by genetic and environmental factors. This comprehensive analysis showcases the distinctions in age, environment, sex, age at diagnosis, SMN2 gene copy number, use of feeding tubes, and ventilator dependence among different SMA types (Table 2).

The median age across the entire cohort is 20 years, with a broad range from 9 to 30.75 years, reflecting a diverse group in terms of age. Regarding the environment, most participants reside in urban areas (69.8%), with a slightly higher proportion in the SMA3 group (75%). The study group comprises more males (53.5%), with an exceptionally high percentage in the SMA1 (80%) and SMA2 (65.2%) groups compared to SMA3 (33.3%). While nearly all SMA1 and SMA2 patients possess two copies (100% and 92.3%, respectively), none of the SMA3 patients do, strongly correlating SMN2 copy number with disease type and severity (*p* < 0.0001). The need for feeding tubes is low across the cohort (7.7%) and similar across types. Ventilator usage, however, varies significantly, with all SMA1 patients requiring ventilatory support, contrasting sharply with SMA3, where only 8.3% need such intervention (*p* = 0.0006).

### 3.3. Dimension Score and General Score of SF-36 Questionnaire Stratified by SMA Type

The analysis presented in Table 3 compares scores across different types of SMA utilizing the SF-36 survey. The study investigates whether there are significant differences in these health dimensions among the SMA types for the impact of the disease’s severity on daily functioning and well-being.

The median physical functioning scores were significantly different (*p* = 0.0002) among the groups, with SMA Type 3 patients exhibiting markedly higher functionality than nearly nonexistent scores in Types 1 and 2. This highlights the severe impact of SMA on physical abilities, particularly in its more severe forms (Types 1 and 2).

Scores for role functioning due to physical health and emotional reasons did not significantly differ among the groups. This suggests a uniform impact on role limitations due to health across different SMA types. The dimensions of energy, fatigue, and emotional well-being also showed no significant differences across SMA types. Social functioning and pain, similarly, showed no statistically significant differences.

The general health score varies across the SMA types. SMA Type 1 patients had the highest average score (60.41 ± 16.79), which suggests a relatively better perceived general health compared to SMA Type 2 (48.73 ± 15.68), but this score was comparable to SMA Type 3 (59.82 ± 15.08).

### 3.4. Score of SF-36 Questionnaire Stratified by Environment

The analysis reveals generally better health outcomes in urban areas compared to rural areas, particularly in physical functioning and general health, where urban residents report higher scores (Table 4). Significant differences are observed only in general health (*p* = 0.014), suggesting notable disparities in overall health perceptions between urban and rural settings.

**Figure 1:**
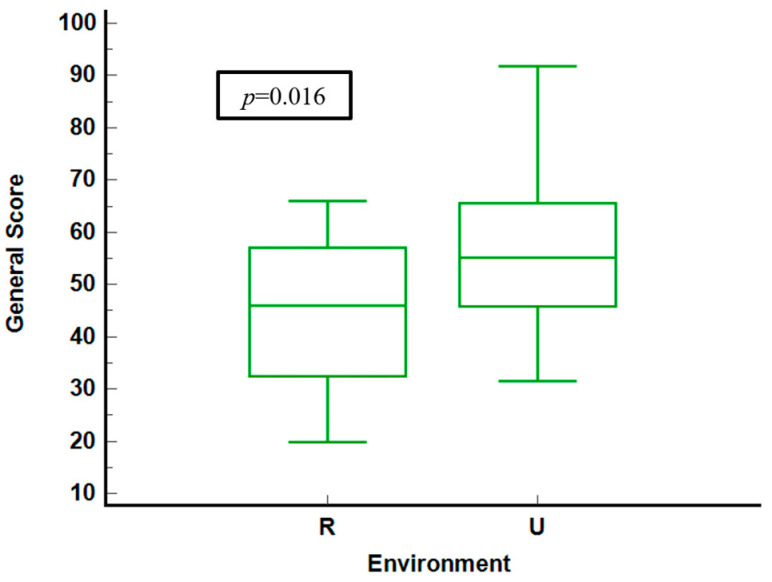
General score stratified by environment. R = rural. U = urban.

Figure 1 compares general health scores of questioned patients in rural and urban environments. Urban residents display higher median scores and a narrower interquartile range (IQR) and lower median scores, indicating more significant variability and poorer health perceptions. The statistical analysis yields a significant *p*-value of 0.016, confirming meaningful differences between the two groups.

**Figure 2:**
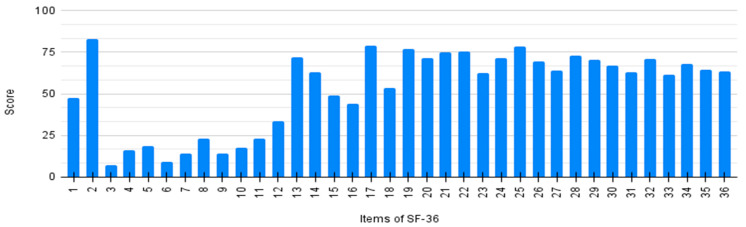
Individual score of SF-36 questionnaire per item.

Every item of SF-36 was individually analyzed by averaging the scores of all patients. Results are detailed in Figure 2. The general score (item 1) shows a significant improvement after the initiation of nusinersen treatment (item 2). Lower scores on items 3–12 and 13–16 demonstrate a poor physical perception related to quality of life.

### 3.5. Score of SF-36 Questionnaire in Correlation with Patients’ Age

#### 3.5.1. General Score Correlated with Age

**Figure 3:**
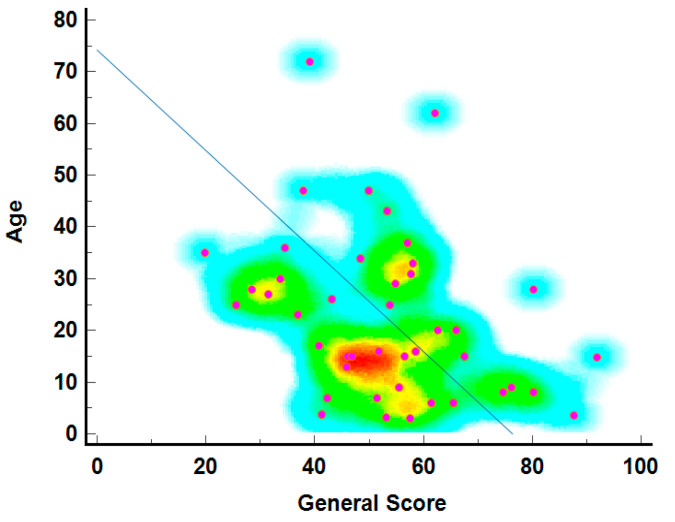
The age–general score correlation heatmap3.5. Individual score of SF-36 questionnaire per Item.

The relationship between age and general health scores is depicted in Figure 3, showing that younger individuals generally report higher general health scores. The density of points, marked by warmer colors (red and yellow), is highest in the 20 to 40 age range with scores between 60 and 80, indicating better-reported health among younger individuals. The diagonal line suggests a theoretical decline in health scores with age, a common trend in QoL studies. Blue and green represent less common age-score combinations, particularly among older individuals with very high or low scores.

#### 3.5.2. SF-36 Score Correlated with Age Groups

To ensure the accuracy of responses, the cohort was divided into two groups: respondents under 14 years old and those over 14. For the younger group, answers were provided by parents or legal guardians (proxy responses), while participants older than 14 responded independently to the questionnaire. This segmentation allowed for more reliable data collection, accommodating respondents’ differing capabilities based on age.

A comparative analysis of health-related QoL dimensions across two age groups, those under 14 and those 14 or older, is provided (Table 5).

No significant differences are observed in physical functioning scores between age groups, with a median score of 5 but differing ranges. Younger participants report better role functioning, particularly in physical and emotional contexts, with a maximum median score compared to older counterparts. Both groups have similar energy levels, though younger participants show slightly higher averages and less variability. A significant difference (*p* = 0.0217) in emotional well-being scores indicates younger individuals perceive a better emotional state, potentially due to parental reporting bias. Younger participants also report significantly better general health (*p* = 0.0072), reflecting higher scores in emotional well-being. The findings suggest younger respondents, or their proxies, report higher health-related QoL, especially in emotional well-being and general health. At the same time, physical and social functioning scores remain consistent across age groups. This highlights the impact of age and reporting mode on perceived health status in QoL assessments.

**Figure 4:**
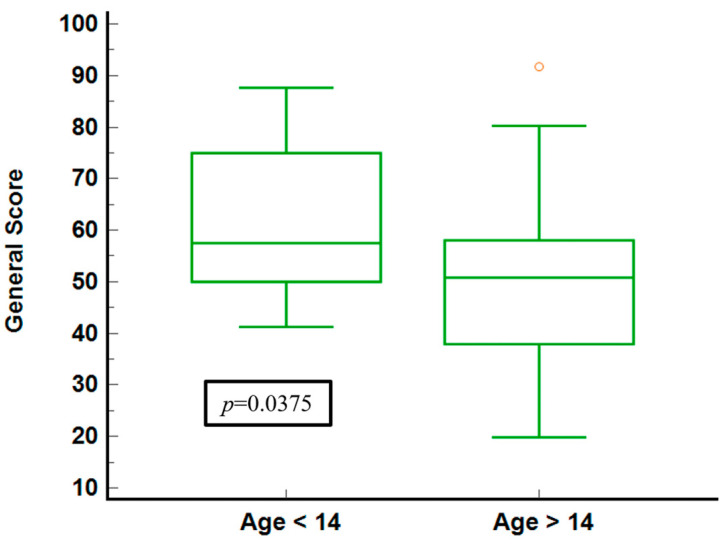
General score stratified by age groups.

The illustration of general health scores for the age groups (Figure 4) shows that younger individuals (under 14) report higher median scores and a narrower IQR, indicating less variability and generally better-perceived health. In contrast, the older group shows a broader IQR and a lower median score, signifying more significant variability and worse health perceptions. The statistical significance (*p* = 0.0375) confirms a meaningful difference between the two age groups.

## 4. Discussion

This study approaches health-related quality of life from a six-month prospective cross-sectional study of 43 Romanian SMA patients treated with nusinersen. This research explores SMA patients’ physical and emotional challenges using the SF-36 Health Survey, with proxy reporting for younger participants. It also critically analyzes nusinersen’s effectiveness in improving patient outcomes and HRQoL, offering valuable insights into managing rare genetic disorders.

HRQoL is defined as the perception of health and daily living status, encompassing physical and mental changes in health and social functioning [30]. HRQoL is assessed with tools like SF-36, which has been extensively validated for reliability in diverse populations, enabling robust comparative and meta-analyses.

Other quality of life assessments include the Pediatric Quality of Life Inventory (PedsQL) [31,32,33], which is specifically designed for children, or the WHOQOL-BREF, which is suitable for adults [34]. Despite its global perspective, WHOQOL-BREF may lack specificity for addressing SMA’s nuanced demands, especially with long-term treatment effects [35]. Though tailored for neurological conditions with modules assessing cognitive function, pain, fatigue, and mental health, Neuro-QoL may not fully capture the everyday living challenges and social complexities integral to SMA [36]. PROMIS is noted for its adaptability, offering detailed evaluations across a broad spectrum of health domains. Nonetheless, its deployment necessitates specific technological resources and comprehensive training [37]. EQ-5D is preferred for efficient, rapid health assessments in extensive studies but may overlook critical health variations in SMA patients [38]. SF-36 was selected for its comprehensive physical and mental health coverage, and because it has been validated across various populations and conditions, including SMA. Its detailed health profiling and robust psychometrics effectively assess SMA’s impact on adults. [39].

The SF-36 Health Survey is a comprehensive, multidimensional, and self-administered questionnaire designed to evaluate perceptions of health-related quality of life across eight domains [28,40]. These domains collectively measure various aspects of an individual’s functional status and well-being, reflecting physical and mental health components [13,41,42]. The SF-36 survey was developed as part of the Rand Corporation’s Health Insurance Experiment in the United States [28], which aimed to appreciate the impact of different health interventions and policies on a population’s health. Subsequently, it was shortened from its original 108-item version [29]. The first version of the SF-36 questionnaire was validated worldwide in 1992 [28] and translated into over 60 languages, including Romanian, as part of the International Quality of Life Assessment Project in 1995 [43]. It was updated in 2012 to Quality Metrics Incorporated—Optum SF-36v2 version, and tested for reliability in the Romanian population in 2019 [44].

In the present study, the cohort’s demographic analysis reveals a right-skewed age distribution, predominantly of younger individuals, with ages ranging widely from young children to middle-aged adults, which is crucial for understanding the population. The average age at diagnosis was 2.953 years, indicating that most diagnoses occur in early childhood, in concordance with most of the published works of the last ten years [3,6,45]. The diagnosis of SMA in early childhood serves as a strong incentive for the implementation of newborn screening programs, as the benefits of early intervention are evident in maintaining muscle function and delaying the onset of severe disability. An evaluation of the four-year utilization of newborn screening in 34 U.S. states reported the diagnosis of 48 newborns with SMA, providing a robust model to follow for better care [46].

Various categorical variables linked to SMA were analyzed, such as the disease type, the number of SMN2 gene copies, geographical location (urban versus rural), feeding tube requirements, and ventilator dependency. Sex distribution varies significantly among the SMA types, indicating potential sex-related genetic or environmental influences on SMA prevalence. Notably, most individuals have Type 2 SMA, followed by Type 3 and Type 1 SMA. Significant deviations from the expected norms indicate differing degrees of prevalence than those reported in the literature [7,8,9]. We must consider that small cohorts of patients characterize research in rare disorders, which can influence the estimation of prevalence. Morcov et al. describe a different cohort of 33 Romanian SMA patients in which Type 2 was the most prevalent, but not the majority, closely followed by Types 1 and 3 [27]. The study also illustrates a stark contrast in the number of SMN2 gene copies, especially between Types 2 and 3, linking gene copy numbers closely with disease type and severity, as shown in the literature [5,6].

The assessment of SMN2 copy number, a prerequisite for patient inclusion in the government-subsidized nusinersen treatment, was uniformly performed in a designated laboratory for all study participants, facilitated by vouchers that allowed for complimentary analysis. Urban residents are overrepresented in the dataset but with no significant statistical differences, suggesting a uniform distribution across environments. On the other hand, the education level of the parents of children affected by SMA may jeopardize the awareness of the disease’s symptoms. Another known issue is the limited disease awareness among doctors, which could delay the diagnosis of SMA. These findings are supported by another Romanian group, indicating a possible clustering of patients based on healthcare services [27]. In general, urban residents in Romania have better access to healthcare resources, with 90.9% of hospitals and hospital-like establishments located in urban areas [47]. Many participants are non-ambulatory, highlighting SMA’s profound impact on physical mobility. Clinical interventions such as feeding tubes show low overall utilization, while ventilator use is highly variable across types, indicating the progressive nature of respiratory challenges in SMA [6]. Mobility restrictions also differ significantly, particularly between Types 2 and 3, underscoring the disease’s physical limitations, particularly in its more severe forms. Overall, characteristics of the dataset stratified by SMA Type 1, Type 2, and Type 3 highlight some differences in clinical presentations and treatment needs shaped by genetic and environmental factors.

The analysis highlights how SMA severity affects patient well-being and daily function. Those with milder SMA report better physical functioning, indicating more mobility and independence than those with severe forms. However, despite physical differences, areas like role functioning, energy, and emotional well-being show slight variation across SMA types, suggesting a uniform impact on patient roles and emotions, possibly due to resilience or coping strategies. General health, social functioning, and pain experiences also remain consistent across types, reflecting a common perception regardless of severity. These insights emphasize the need for health interventions tailored to both physical and psychological aspects to improve life quality for SMA patients [13,32,48,49].

The scores stratified by geographical settings reveal a pattern where urban residents experience better health outcomes, particularly in physical functioning and general health. Urban environments are associated with higher health scores, indicating less variability in health perceptions among individuals, suggesting a more consistent quality of life. In contrast, rural residents show more significant variability in their health scores, generally indicating poorer health perceptions.

Scores of the SF-36 survey reveal a relationship between age and perceived health quality, with distinct differences between younger individuals (under 14 years) and those over 14.

Physical functioning differs significantly from emotional well-being and health perceptions in SMA patients, showing reduced HRQoL in SF-36 studies. SF-36 has been employed in SMA research in various countries, facilitating cross-cultural comparisons and contributing to a global understanding of the disease. Studies from Europe, North America, and Asia have utilized the SF-36 survey to assess HRQoL in SMA patients, highlighting universal challenges and region-specific issues [3,8,13,22,23,36,40].

Younger participants, represented by proxy responses from parents or legal guardians, consistently report higher or more favorable health scores. This trend is particularly evident in physical and emotional well-being and general health measures. As early as 2003, a group from the U.S. conducted interesting research involving care providers who administered Likert-scale surveys on six quality of life measures. The study revealed that, despite the widespread perception that children with SMA1 have a poor quality of life, their care providers do not share this view [48]. They also underscore the potential influence of developmental stages on health perceptions and the effectiveness of health interventions to improve quality of life. While younger children may have fewer health issues, proxy reporting could also skew perceptions, emphasizing a more positive outlook than the actual case [50,51,52]. However, a German pilot study on 17 children with SMA that evaluated them directly through KIDSCREEN-27, KINDL, and PedsQL 3.0 showed significant physical disability but surprisingly good HRQoL as assessed using KIDSCREEN-27. The disease burden was generally higher among non-sitters compared with walkers and SMA Type 1 compared with Type 3 [53]. The study highlights a consistent perception of quality of life among children with SMA and their proxies, which may indicate a proxy bias or truly fewer health issues in early life. This raises questions about the importance of age and whether the respondent is self-reporting or a proxy when interpreting quality of life data. A comparative analysis could clarify these implications. Moreover, Morcov et al.’s use of the PedsQL-Family Impact Module revealed that most children with SMA, as reported by their mothers, did not suffer from significant physical issues like fatigue, headaches, weakness, or stomach problems, nor from emotional difficulties such as anxiety, sadness, anger, frustration, and feelings of despair [27].

The older group, who responded independently, exhibited a broader range of scores and generally perceives their health quality as lower, particularly in general health and physical and emotional role functioning. A literature review could only identify a few studies on the HRQoL of adult patients with SMA before discovering treatment options. However, the quality of life among adult patients can vary widely depending on the type and severity of the disease, the availability of treatments, and individual coping mechanisms. A study from the Netherlands of 62 adult patients with SMA who completed physical component scores and mental component scores from the SF-36 survey revealed that patients with milder forms of SMA tend to have reduced mental QoL [40]. This variance could reflect a more accurate self-assessment of health, possibly recognizing and reporting more health issues as they age.

Interestingly, both age groups show similar physical and social functioning levels, suggesting that essential physical capacities and social interactions are maintained across ages despite differences in overall health perception. The lack of significant differences in physical functioning across the two groups points to a stable physical capacity that does not markedly decline with age in the studied cohort.

As a final remark, our study found that patients with milder forms of SMA, like Types 2 and 3, reported better physical functioning compared to Type 1, suggesting greater mobility and independence in daily activities. However, emotional well-being and general health perceptions remained similar across all types, indicating a consistent impact on their roles and emotional states despite differing physical capabilities.

When examining the quality of life in patients treated with nusinersen, our cohort shows an overall improvement, regardless of age, consistent with expectations. This result aligns with positive outcomes reported in many SMA cohorts, showing improved life quality following treatment [11,12,13,17,23,53]. However, one study contradicted these findings, not demonstrating an improvement in health-related QoL for adult SMA patients as measured by the Neuro-QoL for upper and lower extremity function [36].

Our research holds particular significance as it provides insights into the perception of quality of life among Romanian patients with SMA, encompassing children and adults, reported directly and through proxies. However, the study faces limitations such as a small sample size typical of rare disorders and a geographic focus limited to urban centers in Romania, which may restrict a comprehensive understanding of the broader SMA patient population in Romania. Another weakness of the study is the absence of a control group, which can lead to misinterpretation of results relative to the natural progression of the disease. Moreover, potential biases associated with proxy reporting must be carefully considered.

Future research should broaden its scope to encompass diverse populations and larger cohorts, facilitating direct comparisons between proxy-reported and self-reported outcomes to improve overall reliability.

## 5. Conclusions

This research on Romanian patients with SMA underscores the significance of emotional well-being in shaping health-related quality of life following treatment with nusinersen. Additionally, it revealed significant variations in health perceptions among different age groups, with younger individuals reporting higher quality of life scores compared to their older counterparts. This disparity could be attributed to an optimistic bias in proxy responses and inherent differences in health challenges faced across various age brackets. It emphasizes the critical need for healthcare interventions tailored to specific age groups to effectively address the unique needs of each demographic segment in SMA care.

## Figures and Tables

**Table 1:** Frequency of categorical parameters in the dataset of 43 patients with SMA.

Variable	Level	N	%
SMA Type	1	5	11.6
	2	26	60.4
	3	12	27.9
Number of SMN2 copy	2	29	67.4
	3	14	32.6
Urban/Rural	R	13	30.2
	U	30	69.8
Tube feeding = 1 Normal feeding = 0	0	41	95.3
	1	2	4.7
Ventilator = 1 No respiratory assistance = 0	0	21	48.8
	1	22	51.2

N = number of patients.

**Table 2:** Comparative analysis of clinical and demographic characteristics across SMA types.

	Total	SMA1 n = 5	SMA2 n = 26	SMA3 n = 12	*p*-Value
Age (M + IQR)	20 (9–30.75)	3.58 (3.8–6.25)	20 (15–33)	26.5 (14.5–33.5)	0.0023 ^1^
Environment—urban (M + IQR)	30 (69.8%)	3 (60%)	18 (69.2%)	9 (75%)	0.82 ^1^
Sex—male (M + IQR)	23 (53.5%)	4 (80%)	15 (65.2%)	4 (33.3%)	0.168 ^1^
Age at diagnosis (M + IQR)	2 (2–3)	1 (1–1)	2 (2–2)	5 (3–8.5)	<0.0001 ^1^
SMN 2 copies—2 (M + IQR)	29 (67.4%)	5 (100%)	24 (92.3%)	0 (0%)	<0.0001 ^1^
Feeding tube—yes (M + IQR)	2 (4.7%)	0	2 (7.7%)	0	0.5 ^1^
Ventilator—yes (M + IQR)	22 (51.2%)	5 (100%)	16 (61.5%)	1 (8.3%)	0.0006 ^1^

^1^ Mann–Whitney. n = number of patients.

**Table 3:** SF-36 dimensions scores across SMA types.

	Total	SMA1 n = 5	SMA2 n = 26	SMA3 n = 12	*p*-Value
Physical functioning (M + IQR)	5 (0–23.75)	0 (0–20)	0 (0–10)	50 (15–60)	0.0002 ^1^
Role functioning/physical (M + IQR)	50 (25–100)	100 (18.75–100)	50 (0–100)	75 (50–100)	0.348 ^1^
Role functioning/emotional (M + IQR)	66.67 (33.33–100)	100 (91.66–100)	83.33 (33.33–100)	66.66 (50–100)	0.255 ^1^
Energy/fatigue (M + IQR)	65 (19.71)	69 (16.35)	64.61 (21.16)	64.16 (18.92)	0.839 ^1^
Emotional well-being (M + IQR)	72 (64–88)	84 (70–97)	74 (64–88)	62 (56–88)	0.275 ^1^
Social functioning (M + IQR)	75 (50–100)	87.5 (59.37–90.62)	75 (50–100)	75 (37.5–93.75)	0.85 ^1^
Pain (M + IQR)	77.5 (55.62–100)	100 (86.25–100)	77.5 (55–100)	78.75 (56.25–100)	0.38 ^1^
General health (A + SD)	61.04 (20.45)	77 (5.7)	58.84 (21.64)	59.16 (19.75)	0.181 ^2^
General score (A + SD)	53.18 (16.24)	60.41 (16.79)	48.73 (15.68)	59.82 (15.08)	0.082 ^2^

^1^ Mann–Whitney. ^2^ *t*-test. n = number of patients.

**Table 4:** Analysis of scores by environment.

	Total	Urban	Rural	*p*-Value
Physical functioning (M + IQR)	5 (0–23.75)	7.5 (0–40)	0 (0–12.5)	0.141 ^1^
Role functioning/physical (M + IQR)	50 (25–100)	75 (25–100)	50 (0–81.25)	0.311 ^1^
Role functioning/emotional (M + IQR)	66.67 (33.33–100)	100 (33.33–100)	66.66 (33.33–100)	0.216 ^1^
Energy/fatigue (A + SD)	65 (19.71)	68.83 (16.27)	56.15 (24.42)	0.051 ^2^
Emotional well-being (M + IQR)	72 (64–88)	76 (64–92)	64 (54–79)	0.102 ^1^
Social functioning (M + IQR)	75 (50–100)	75 (50–100)	75 (37.5–87.5)	0.21 ^1^
Pain (M + IQR)	77.5 (55.62–100)	88.75 (67.5–100)	77.5 (32.5–100)	0.216 ^1^
General health (A + SD)	61.04 (20.45)	66 (18.11)	49.61 (21.64)	0.014 ^2^

^1^ Mann–Whitney. ^2^ *t*-test.

**Table 5:** Comparative analysis of health-related QoL dimensions across two age groups.

	Total	Age < 14	Age ≥ 14	*p*-Value
Physical functioning (M + IQR)	5 (0–23.75)	5 (0–45)	5 (0–20)	0.608 ^1^
Role functioning/physical (M + IQR)	50 (25–100)	100 (25–100)	50 (0–100)	0.147 ^1^
Role functioning/emotional (M + IQR)	66.67 (33.33–100)	100 (66.66–100)	66.67 (33.33–100)	0.155 ^1^
Energy/fatigue (A + SD)	65 (19.71)	68.46 (13.59)	63.5 (21.85)	0.454 ^2^
Emotional well-being (M + IQR)	72 (64–88)	84 (70–96)	68 (60–88)	0.0217 ^1^
Social functioning (M + IQR)	75 (50–100)	75 (59.37–90.62)	75 (50–100)	0.756 ^1^
Pain (M + IQR)	77.5 (55.62–100)	100 (71.87–100)	77.5 (55–100)	0.331 ^1^
General Health (A + SD)	61.04 (20.45)	73.46 (13.28)	55.66 (20.83)	0.0072 ^2^

^1^ Mann–Whitney. ^2^ *t*-test.

## Data Availability

The data presented in this study are available upon request from the corresponding author. Due to ethical restrictions, they are not publicly available.

## References

[1] Werdnig G. (1971). Two early infantile hereditary cases of progressive muscular atrophy simulating dystrophy, but on a neural basis. 1891. Arch. Neurol..

[2] Hoffmann J. (1893). Ueber chronische spinale Muskelatrophie im Kindesalter, auf familiärer Basis. Dtsch. Z. Nervenheilkd..

[3] Nishio H., Niba E.T.E., Saito T., Okamoto K., Takeshima Y., Awano H. (2023). Spinal Muscular Atrophy: The Past, Present, and Future of Diagnosis and Treatment. Int. J. Mol. Sci..

[4] Milligan J.N., Blasco-Pérez L., Costa-Roger M., Codina-Solà M., Tizzano E.F. (2022). Recommendations for Interpreting and Reporting Silent Carrier and Disease-Modifying Variants in SMA Testing Workflows. Genes.

[5] Monani U.R., Lorson C.L., Parsons D.W., Prior T.W., Burghes A.H., McPherson J.D. (1999). A single nucleotide difference that alters splicing patterns distinguishes the SMA gene SMN1 from the copy gene SMN2. Hum. Mol. Genet..

[6] Keinath M.C., Prior D.E., Prior T.W. (2021). Spinal Muscular Atrophy: Mutations, Testing, and Clinical Relevance. Appl. Clin. Genet..

[7] Darras B.T. (2015). Spinal muscular atrophies. Pediatr. Clin. N. Am..

[8] Arnold W.D., Kassar D., Kissel J.T. (2015). Spinal muscular atrophy: Diagnosis and management in a new therapeutic era. Muscle Nerve.

[9] Kolb S.J., Kissel J.T. (2015). Spinal Muscular Atrophy. Neurol. Clin..

[10] Zerres K., Rudnik-Schöneborn S. (1995). Natural history in proximal spinal muscular atrophy. Clinical analysis of 445 patients and suggestions for a modification of existing classifications. Arch. Neurol..

[11] Nevo Y., Wang C. (2016). Spinal muscular atrophy: A preliminary result toward new therapy. Neurology.

[12] Ramdas S., Oskoui M., Servais L. (2024). Treatment Options in Spinal Muscular Atrophy: A Pragmatic Approach for Clinicians. Drugs.

[13] AlRuthia Y., Almuaythir G.S., HAlrasheed H., Alsharif W.R., Temsah M.-H., Alsohime F., Sales I., Alwhaibi M., Bashiri F.A. (2021). Proxy-Reported Quality of Life and Access to Nusinersen Among Patients with Spinal Muscular Atrophy in Saudi Arabia. Patient Prefer. Adherence.

[14] Arslan D., Inan B., Kilinc M., Bekircan-Kurt C.E., Erdem-Ozdamar S., Tan E. (2023). Nusinersen for adults with spinal muscular atrophy. Neurol. Sci..

[15] Aslesh T., Yokota T. (2022). Restoring SMN Expression: An Overview of the Therapeutic Developments for the Treatment of Spinal Muscular Atrophy. Cells.

[16] Belter L., Cruz R., Jarecki J. (2020). Quality of life data for individuals affected by spinal muscular atrophy: A baseline dataset from the Cure SMA Community Update Survey. Orphanet J. Rare Dis..

[17] Bonanno S., Zanin R., Bello L., Tramacere I., Bozzoni V., Caumo L., Ferraro M., Bortolani S., Sorarù G., Silvestrini M. (2022). Quality of life assessment in adult spinal muscular atrophy patients treated with nusinersen. J. Neurol..

[18] Chambers G.M., Settumba S.N., Carey K.A., Cairns A., Menezes M.P., Ryan M., Farrar M.A. (2020). Prenusinersen economic and health-related quality of life burden of spinal muscular atrophy. Neurology.

[19] Dangouloff T., Hiligsmann M., Deconinck N., D’Amico A., Seferian A.M., Boemer F., Servais L. (2023). Financial cost and quality of life of patients with spinal muscular atrophy identified by symptoms or newborn screening. Dev. Med. Child. Neurol..

[20] Holm A., Hansen S.N., Klitgaard H., Kauppinen S. (2022). Clinical advances of RNA therapeutics for treatment of neurological and neuromuscular diseases. RNA Biol..

[21] Lakhina Y., Boulis N.M., Donsante A. (2023). Current and emerging targeted therapies for spinal muscular atrophy. Expert. Rev. Neurother..

[22] Weaver M.S., Hanna R., Hetzel S., Patterson K., Yuroff A., Sund S., Schultz M., Schroth M., Halanski M.A. (2020). A Prospective, Crossover Survey Study of Child- and Proxy-Reported Quality of Life According to Spinal Muscular Atrophy Type and Medical Interventions. J. Child. Neurol..

[23] Mix L., Winter B., Wurster C.D., Platen S., Witzel S., Uzelac Z., Graf H., Ludolph A.C., Lule D. (2021). Quality of Life in SMA Patients under Treatment with Nusinersen. Front. Neurol..

[24] Qiu J., Wu L., Qu R., Jiang T., Bai J., Sheng L., Feng P., Sun J. (2022). History of development of the life-saving drug “Nusinersen” in spinal muscular atrophy. Front. Cell Neurosci..

[25] Signoria I., van der Pol W.L., Groen E.J.N. (2023). Innovating spinal muscular atrophy models in the therapeutic era. Dis. Model. Mech..

[26] Şimşek Erdem N., Güneş Gencer G.Y., Alaamel A., Uysal H. (2024). Effect of nusinersen treatment on quality of life and motor function in adult patients with spinal muscular atrophy. Neuromuscul. Disord..

[27] Morcov M.V., Padure L., Morcov C.G., Onose G. (2021). Findings regarding emotion regulation strategies and quality of life’s domains in families having children with spinal muscular atrophy. J. Med. Life.

[28] Brazier J.E., Harper R., Jones N.M., O’Cathain A., Thomas K.J., Usherwood T., Westlake L. (1992). Validating the SF-36 health survey questionnaire: New outcome measure for primary care. BMJ.

[29] Mchorney C.A., Johne W., Anastasiae R. (1993). The MOS 36-Item Short-Form Health Survey (SF-36): II. Psychometric and Clinical Tests of Validity in Measuring Physical and Mental Health Constructs. Medical Care.

[30] Yin S., Njai R., Barker L., Siegel P.Z., Liao Y. (2016). Summarizing health-related quality of life (HRQOL): Development and testing of a one-factor model. Popul. Health Metr..

[31] Klug C., Schreiber-Katz O., Thiele S., Schorling E., Zowe J., Reilich P., Walter M.C., Nagels K.H. (2016). Disease burden of spinal muscular atrophy in Germany. Orphanet J. Rare Dis..

[32] Kölbel H., Modler L., Blaschek A., Schara-Schmidt U., Vill K., Schwartz O., Müller-Felber W. (2022). Parental Burden and Quality of Life in 5q-SMA Diagnosed by Newborn Screening. Children.

[33] Vaidya S., Boes S. (2018). Measuring quality of life in children with spinal muscular atrophy: A systematic literature review. Qual. Life Res..

[34] Silva P.A.B., Soares S.M., Santos J.F.G., Silva L.M. (2014). Cut-off point for WHOQOL-bref as a measure of quality of life of older adults. Rev. Saúde Pública.

[35] Geng D., Ou R., Miao X., Zhao L., Wei Q., Chen X., Liang Y., Shang H., Yang R. (2017). Patients’ self-perceived burden, caregivers’ burden and quality of life for amyotrophic lateral sclerosis patients: A cross-sectional study. J. Clin. Nurs..

[36] Thimm A., Brakemeier S., Kizina K., Rosales J.M., Stolte B., Totzeck A., Deuschi C., Kleinschnitz C., Hagenacker T. (2021). Assessment of Health-Related Quality of Life in Adult Spinal Muscular Atrophy Under Nusinersen Treatment—A Pilot Study. Front. Neurol..

[37] Crawford T., Day J.W., De Vivo D.C., Kruger J.M., Mercuri E., Nascimento A., Pasternak A., Mazzone E. S., Duong T., Song G. (2024). Long-term efficacy, safety, and patient-reported outcomes of apitegromab in patients with spinal muscular atrophy: Results from the 36-month TOPAZ study. Front. Neurol..

[38] López-Bastida J., Peña-Longobardo L.M., Aranda-Reneo I., Tizziano E., Sefton M., Olivia-Moreno J. (2017). Social/economic costs and health-related quality of life in patients with spinal muscular atrophy (SMA) in Spain. Orphanet J. Rare Dis..

[39] Messina S., Sframeli M. (2020). New Treatments in Spinal Muscular Atrophy: Positive Results and New Challenges. J. Clin. Med..

[40] Kruitwagen-Van Reenen E.T., Wadman R.I., Visser-Meily J.M., Van den Berg L.H., Schröder C., Ludo van der Pol W. (2016). Correlates of health related quality of life in adult patients with spinal muscular atrophy. Muscle Nerve.

[41] Landfeldt E., Udo C., Lövgren M., Sejersen T., Kreicbergs U. (2023). Health-related quality of life of children with spinal muscular atrophy in Sweden: A prospective cohort study in the era of disease-modifying therapy. Eur. J. Paediatr. Neurol..

[42] Yang M., Awano H., Tanaka S., Toro W., Zhang S., Dabbous O., Igarashi A. (2022). Systematic Literature Review of Clinical and Economic Evidence for Spinal Muscular Atrophy. Adv. Ther..

[43] Scoggins J.F., Patrick D.L. (2009). The Use of Patient-Reported Outcomes Instruments in Registered Clinical Trials: Evidence from ClinicalTrials.gov. Contemp. Clin. Trials.

[44] Mardare I., Furtunescu F.L., Bratu E.C. (2019). Measuring health related quality of life—Methods and tools. Acta Medica Transilv..

[45] Mazzella A., Curry M., Belter L., Cruz R., Jarecki J. (2021). “I have SMA, SMA doesn’t have me”: A qualitative snapshot into the challenges, successes, and quality of life of adolescents and young adults with SMA. Orphanet J. Rare Dis..

[46] Hale K., Ojodu J., Singh S. (2021). Landscape of Spinal Muscular Atrophy Newborn Screening in the United States: 2018–2021. Int. J. Neonatal Screen..

[47] Petre I., Barna F., Gurgus D., Tomescu L.C., Apostol A., Petre I., Furau C., Năchescu M.L., Bordianu A. (2023). Analysis of the Healthcare System in Romania: A Brief Review. Healthcare.

[48] Bach J.R., Vega J., Majors J., Friedman A. (2003). Spinal muscular atrophy type 1 quality of life. Am. J. Phys. Med. Rehabil..

[49] Lloyd A.J., Thompson R., Gallop K., Teynor M. (2019). Estimation of the Quality of Life Benefits Associated with Treatment for Spinal Muscular Atrophy. Clin. Outcomes Res..

[50] Brandt M., Johannsen L., Inhestern L., Bergelt C. (2022). Parents as informal caregivers of children and adolescents with spinal muscular atrophy: A systematic review of quantitative and qualitative data on the psychosocial situation, caregiver burden, and family needs. Orphanet J. Rare Dis..

[51] Aksaralikitsunti M., Sanmaneechai O. (2022). Health-related quality of life in Thai children with spinal muscular atrophy. Pediatr. Neonatol..

[52] Yao M., Ma Y., Qian R., Xia Y., Yuan C., Bai G., Mao S. (2021). Quality of life of children with spinal muscular atrophy and their caregivers from the perspective of caregivers: A Chinese cross-sectional study. Orphanet J. Rare Dis..

[53] Landfeldt E., Leibrock B., Hussong J., Thiele S., Abner S., Walter M.C., Moehler E., Zemlin M., Dillmann U., Flotats-Bastardas M. (2024). Self-Reported Health-Related Quality of Life of Children with Spinal Muscular Atrophy: Preliminary Insights from a Nationwide Patient Registry in Germany. J. Neuromuscul. Dis..

